# Nocturnal foliar water uptake: an unexplored process in crops?

**DOI:** 10.1093/jxb/eraf524

**Published:** 2025-12-02

**Authors:** Lorna McAusland, Andrew Smith, Erik Murchie

**Affiliations:** Division of Plant and Crop Sciences, School of Biosciences, University of Nottingham, Leicestershire LE12 5RD, UK; British Geological Survey, Keyworth, Nottingham NG12 5GG, UK; Division of Plant and Crop Sciences, School of Biosciences, University of Nottingham, Leicestershire LE12 5RD, UK; University of Illinois Urbana-Champaign, USA

**Keywords:** Dew, foliar uptake, humidity, leaves, night, stomata, stomatal conductance, *Triticum aestivum*, water, wheat

**Plant water uptake is commonly rationalized as a daytime, root-driven process, mediated by stomatal responses to minimize water loss, optimize photosynthesis, and promote growth. This has led to a certain experimental bias in favor of studying daytime mechanisms and their role in survival under climate change (Gaston, 2019). However, critical, physiological processes also occur nocturnally and respond to changing climates. Despite advances into hydraulics, nocturnal foliar mechanisms facilitating crop survival under rising nighttime temperatures and water scarcity remain understudied. Here we highlight sources of nocturnal foliar water and discuss the leaf-level processes which could underpin crop resilience to warmer nights (Caird *et al*., 2007; Schoppach *et al*., 2014).**

## Nocturnal water availability plays a vital role in plant–environment interactions

Nocturnal water flux through the plant contributes substantially to total daily water budget and supports primary productivity and terrestrial ecosystems, but until recently, was not accounted for in crop models (Schoppach *et al*., 2014). Typified by transitions between high daytime to low nighttime temperatures, windspeeds, and vapor pressure deficits (VPD), nighttime environmental conditions play a distinct, integral role in plant survival. As climate change and anthropogenic activities continue, plant responses to changing nocturnal environments are becoming more important. Nights are warming more rapidly than days (Cox *et al*., 2020), leading to changes in primary processes such as increased plant respiration that not only reduces growth and yield (Posch *et al*., 2022; McAusland *et al*., 2023), but also decreases available water and leaf wetting events.

## Dew and fog represent a nocturnal water source for foliar water uptake in above-ground tissues

**Fig. 1.**
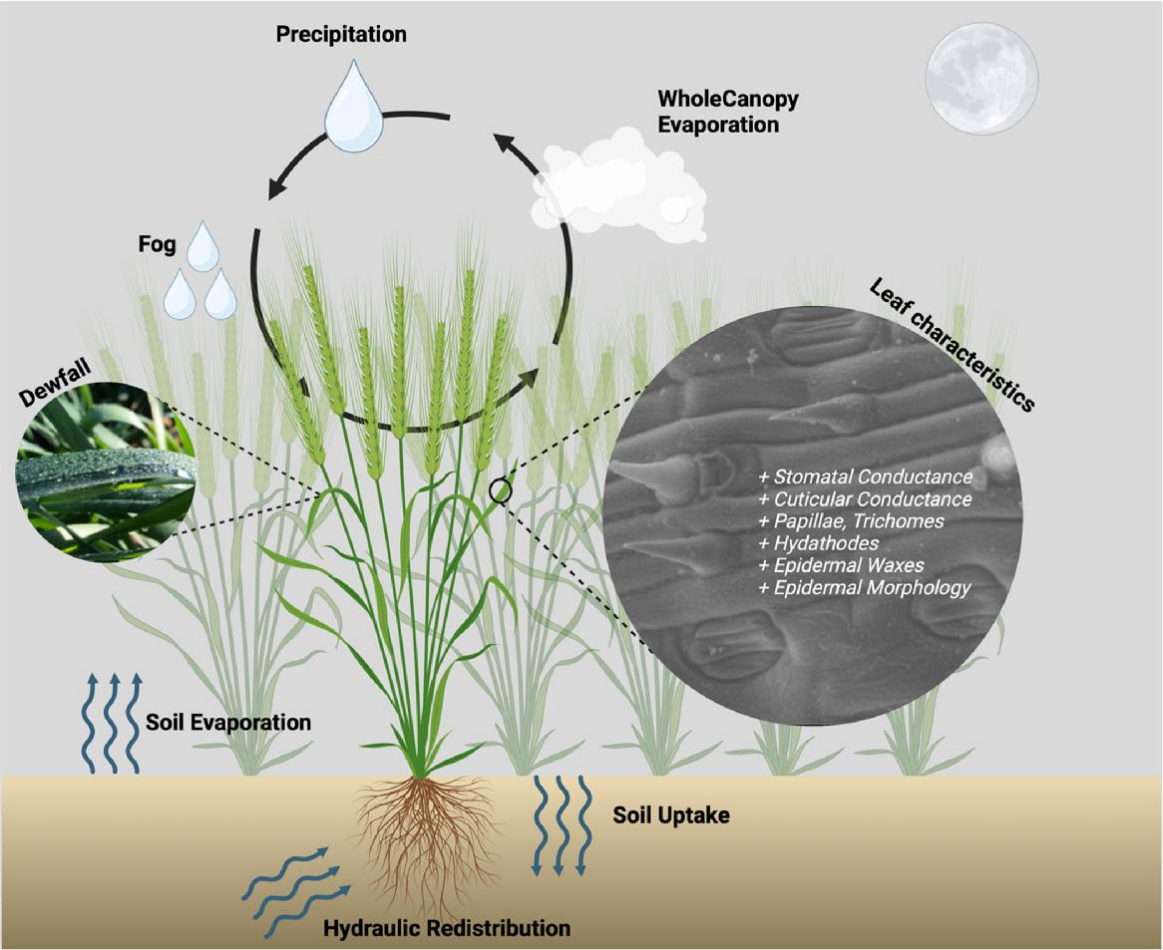
Visualizing the interaction of a crop plant with nocturnal environment. In contrast to high daytime temperatures, low nocturnal temperatures, low vapor pressure deficits and windspeeds support the formation of dew on leaf surfaces. Dew, fog and precipitation contribute to the available water for the plant via two pathways: (i) water absorbed by the soil and taken up by the roots either locally or by hydraulic redistribution; or (ii) via contact with above-ground organs such as leaves. Surface water cools leaves but also provides a water source for foliar water uptake. Structures on the leaf, including stomata, epidermal morphology and trichomes may support uptake of water at night. Warm, dry environmental conditions exacerbated by climate change promote canopy water loss in the form of whole canopy evaporation, reducing surface water available for localized rehydration. Created in BioRender. McAusland, L. (2025) https://BioRender.com/4a0sbpb.

Nocturnal leaf wetting events including precipitation (e.g. rainfall, sleet, and snow), mist, fog, and dewfall are estimated to occur up to 100–200 days per year across ecosystems worldwide (Dawson and Goldsmith, 2018), therefore representing a significant source of available water for plants, particularly those growing in dry seasons or semi-arid/arid regions (Fig. 1). Indeed, dew was shown to be vital for plant growth in some functional types (Feng *et al*., 2021). While precipitation describes droplets heavy enough to fall by gravity, mist (∼100 μm in diameter) and fog (∼10–50 μm in diameter) are suspended in the atmosphere. In contrast, dew occurs when a surface temperature is lower or equal to the dewpoint, causing water to condense on that surface in droplets. Typical dew yields range between 0.01–0.3 mL m^-2^ night^−1^, but have been observed as high as 0.6 L m^−2^ night^−1^ (Beysens, 2022). Nocturnal cloud cover, distance to the coast, windspeed and ambient temperature, all play a role in the severity and duration of the dew yield.

Dew that forms on the plant canopy can potentially enter the leaves through three pathways: uptake through the cuticle, uptake from specialized structures on the epidermis (e.g. specialized epidermal structures such as hydrophilic trichomes, papillae, or hydathodes), or entering via the stomata. While water deposition on plant aerial surfaces can promote onset of biotic stresses (e.g. establishment of fungal diseases) and potential deposition of pollutants, it also represents an important source of water availability in the form of foliar water uptake (FWU) (Dawson and Goldsmith, 2018).

## What leaf characteristics facilitate foliar water uptake in crops?

FWU has been shown to occur in trees, succulent or desert species, and shrubs (Schreel and Steppe, 2020), with water entering via the stomata, cuticle (Guzmán-Delgado *et al*., 2021), hydrophilic trichomes (Fernández and Eichert, 2009), and hydathode pores (Martin and von Willert, 2000). Within the leaf, the presence of aquaporins—specialized channels within cell membranes regulating water movement—have also been linked to FWU (Yan *et al*., 2015).

Many of these species also display nocturnal stomatal opening (*g*_sn_; Caird *et al*., 2007) which provides access into the plant under conditions of high humidity (e.g. fog) or dewfall. However, FWU has not been shown to occur in crop species such as wheat (*Triticum aestivum*). Wheat is our most widely grown crop, covering 218 million hectares world-wide and inhabiting many different growing environments: from cool and wet, to warm and arid (Gbegbelegbe *et al*., 2017). Hot, clear days and cool nights lead to heavy nocturnal dewfall on the crop canopy (Fig. 1).

Our recent work has shown that, like trees and succulents, Mexican wheat cultivars open their stomata at night (McAusland *et al*., 2021, 2023). Traditionally, *g*_sn_ has been thought of as water-wasteful in agriculture; however it has not been possible to test trade-offs empirically. In wheat, *g*_sn_ accounts for up to 18% of daytime rates of water loss from stomata (McAusland *et al*., 2023). It remains conceivable that *g*_sn_ could be utilized by wheat cultivars to retain canopy surface water in semi-arid or arid conditions via FWU. Despite wheat having a wide agroecological range and hence exposure to varied nocturnal conditions, data documenting the duration and intensity of dew on wheat canopies is scarce. Methods such as dew harvesting and leaf wetness sensors could be more readily employed to quantify the variability in fog and dew events (Tashtoush and Alshoubaki, 2023).

**Fig. 2.**
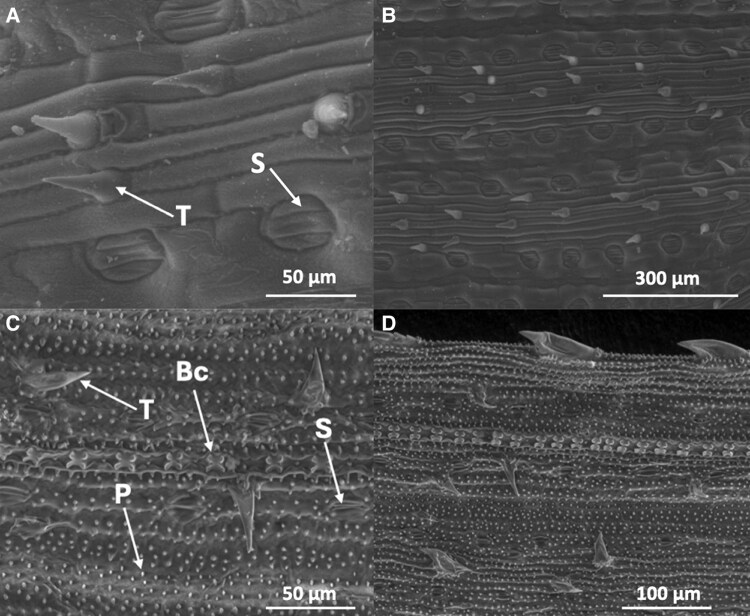
The complexity of leaf surface structures. (A)The adaxial surface of a tillering wheat leaf at 1000× and (B) 250× magnification and seedling rice leaf at (C) 1000× (adaxial surface) and (D) 500× magnification (abaxial surface). For the wheat leaf, the stomata (S) are clearly arranged in rows running parallel to rows of epidermal cells, interspersed with trichomes (T). The furrows formed by the epidermis could influence the movement of condensed water from dewfall. For the rice leaf, stomata are similarly arranged in rows alongside bulliform cells (Bc) with the trichomes interspersed with numerous epidermal papillae (P). Size of scale bars is shown.

Although stomatal pores provide an obvious opening facilitating FWU (Guzmán-Delgado *et al*., 2021), it is not the only possible route. Cuticle conductance, trichomes, and water entering via hydathodes also provide potential pathways for foliar water uptake in wheat, albeit on a smaller scale. Hydathodes are specialized pores on leaf margins and usually thought of as the route for extrusion of liquid water during guttation. However, they are also used by some species to absorb dew at night, aided by parenchyma tissue with direct connection to vascular tissue (Jauneau *et al*., 2020), and are present in wheat (Maeda and Maeda, 1987). There is evidence that trichomes can drive FWU in epiphytes and tree species (Schreel *et al*., 2020). However, there is significant room for discovery here, because many leaf surfaces possess diverse structures, such as rice papillae that have been shown to influence disease resistance and water use efficiency (Pitaloka *et al*., 2021), and may influence physical surface properties (Fig. 2).

Often also referred to interchangeably as minimum conductance (cuticular conductance plus incomplete stomatal closure), cuticular conductance is an order of magnitude smaller than *g*_sn_, ranging between 2–10 mmol m^−2^ s^−1^, and levels have been shown to be genotype- and organ-specific (Araus *et al*., 1991; Duursma *et al.*, 2019; McAusland *et al.*, 2021).

**Fig. 3.**
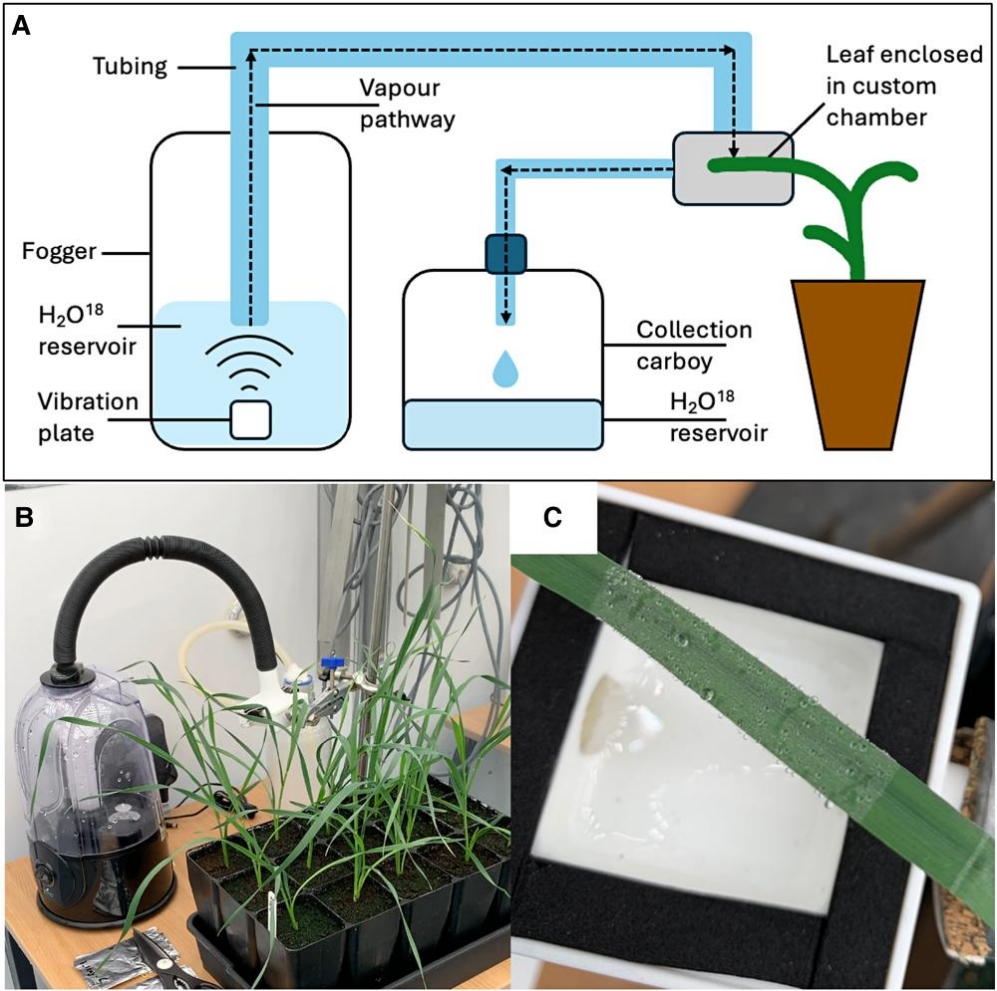
Testing the foliar uptake of water by leaves. (A) A schematic depiction of the setup for treatment of wheat leaves with vaporized oxygen-18 enriched water. (B) The water is vaporized using high frequency vibration plate, located in a commercial terrarium fogger located within a controlled environment room. (C) The misted water falls on the leaf sample, forming ‘dew droplets’—a process otherwise unavailable in controlled growth environments. For plant growth conditions see McAusland *et al*. (2021).

**Fig. 4.**
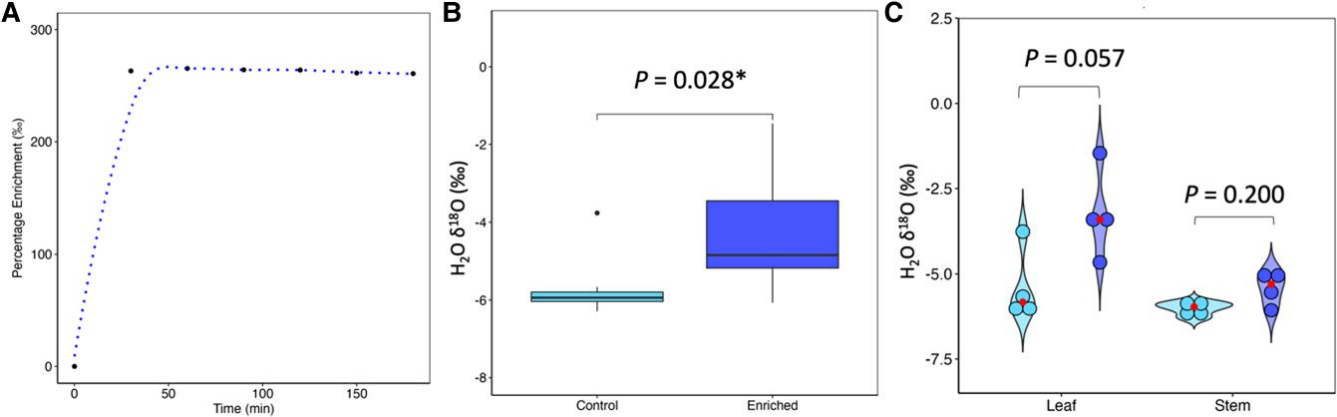
Water is taken up by the leaf from the atmosphere. Oxygen-18 (^18^O) enriched water was fogged into a custom leaf chamber to investigate foliar water uptake in wheat leaves. (A) After ∼25 min of treatment, the direct permille isotope value (δ^18^O-H_2_O—‰) was stable within the treatment chamber for the duration of the experiment. (B) Plant material was sampled for determination of ^18^O enrichment from leaves either fogged with the ^18^O-enriched H_2_O (dark blue) or fogged ddH_2_O (light blue). (C) Finally, to determine whether ^18^O-enriched water was transported from the leaves to other areas of the plant, the permille isotope value was separated into either leaf or stem material from the same tiller. An unpaired *t*-test was used to determine differences between treated (dark blue) and untreated (light blue) samples. The red point (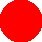) indicates the mean enrichment for each organ; *n*=4–5 biological replicates. The asterisk (*) denotes significant differences (*P*<0.05) according to a *t*-test.

Along with measuring change in water potential or leaf mass, a method for assessing whether dewfall enters the leaf is exposure to artificial dew enriched with a naturally occurring isotope, oxygen-18 (^18^O, Fig. 3). While care should be taken when inferring FWU using stable isotopes (Goldsmith *et al*, 2017), exposing a well-watered leaf to vaporized ^18^O within a custom chamber enables addition of signal which can be identified and analyzed using mass spectroscopy (Fig. 4). Enriching the dew by up to 263‰ (Fig. 4A), enabled detection of a 24% increase in ^18^O in treated plant material of a high *g*_sn_-containing spring wheat genotype (Fig. 4B, C). This demonstrates the potential for water to move into the wheat leaf at night under dew-forming conditions, and be distributed locally.

While there was a significant increase in ^18^O enrichment overall within the tiller (leaf+stem—Fig. 4C) confirming nocturnal water uptake, this experiment does not give insight into the dynamics of distribution, due to the limited sampling points and times.

Currently, the role of FWU in crops such as wheat is unclear. Any water taken up may simply be lost via transpiration in the initial hours of daylight, with estimates suggesting that 10 h of fog absorption would only support limited periods of transpiration (Guzmán-Delgado, 2021). However, early morning stomatal conductance drives an important yield-forming component of photosynthesis (Pinto *et al*., 2024), which may also provide local rehydration and enable maintenance of cell turgidity under water-limiting conditions.

FWU can provide a novel, beneficial role for nocturnal stomatal behavior in crops such as wheat, and generates exciting new breeding traits for a characteristic traditionally associated with ecological studies into trees and extremophiles. As *g*_sn_ is genotype-specific, it is plausible that FWU also has a genetic component which could be targeted to improve resilience for warmer nights and less nocturnal precipitation. This raises an interesting question: by seeking higher yields under an unconscious daytime bias, have we inadvertently lost genetic variation in a critical nocturnal trait?

## Perspectives

Dewfall represents a significant source of water that is independent from soil reserves. While certain plant species have evolved to exploit foliar wetting, it has not been considered for the improvement of crop water budgets. Plant and crop foliar uptake therefore represents a severely under-studied area of research (Box 1), which could uncover novel mechanisms for nocturnal water-uptake when climate change threatens soil water availability.

Box 1. **Outstanding questions**

- Can we improve our methodologies for determining dewfall on crops—both to quantify and to monitor the duration canopies are wet?
- What leaf surfaces promote dewfall capture?
- What is the main route for water uptake: stomata, cuticle, or specialized cells?
- Does nocturnal stomatal conductance occur at significant magnitudes in other crop species?
- Does foliar water uptake play a role in heat or drought tolerance in crops?
- If water is taken up at the leaf via *g*_sn_, does it remain a passive mechanism that maintains humidity in the intracellular spaces, or does it contribute to localized hydration through incorporation in key tissues or cell types?
- Can we prove *g*_sn_ is the sole facilitator of FWU, or is it due to another leaf-level characteristic?
- Water as a carrier for foliar application of chemicals: is it beneficial, such as fertilizers or detrimental, such as pollutants?

## Data Availability

Source data supporting the findings of this study are available from the corresponding author upon reasonable request.
